# Exploring the perspective of adolescent childhood cancer survivors on follow‐up care and their concerns regarding the transition process—A qualitative content analysis

**DOI:** 10.1002/cam4.7234

**Published:** 2024-05-16

**Authors:** Jana Winzig, Laura Inhestern, Désirée Sigmund, Verena Paul, Lesley‐Ann Hail, Stefan Rutkowski, Gabriele Escherich, Corinna Bergelt

**Affiliations:** ^1^ Department of Medical Psychology University Medical Center Hamburg‐Eppendorf Hamburg Germany; ^2^ Department of Pediatric Hematology and Oncology University Medical Center Hamburg‐Eppendorf Hamburg Germany; ^3^ Department of Medical Psychology University Medicine Greifswald Greifswald Germany

**Keywords:** adolescence, childhood cancer survivors, concerns, pediatric follow‐up care, psycho‐oncology, transition process

## Abstract

**Purpose:**

In Germany, children diagnosed with cancer survive their initial disease in more than 80%, and the majority will become long‐term survivors. Around the age of 18, survivors are transferred to adult healthcare. The transition can be a critical period in the process of care at which many childhood cancer survivors discontinue to participate in regular follow‐up care. Hence, the objective of the paper was to explore (a) survivors' attitudes towards pediatric follow‐up care and (b) their concerns regarding the transition process to draw conclusions for optimizing pediatric care and transition processes.

**Methods:**

We conducted semi‐structured interviews with 21 adolescent childhood cancer survivors between the ages of 14 and 20. The survivors were recruited via a pediatric oncology department of a university hospital in Germany. Based on the principles of qualitative content analysis, a deductive‐inductive method according to Kuckartz was applied.

**Results:**

Based on the interview guide and derived from the exploratory research questions, two key categories were generated: (a) Survivors' attitudes towards pediatric follow‐up care, which encompasses all formal and emotional aspects of survivors regarding follow‐up care, and (b) their concerns regarding transition from pediatric to adult healthcare, where hindering and facilitating factors for a successful transition occur. Our results show high satisfaction among survivors with follow‐up care. Nevertheless, they wish to be more integrated into processes and the organization of their follow‐up care. Most adolescent survivors do not feel ready for transition.

**Conclusion:**

The integration of survivors into the organization processes and routines, and the promotion of emotional detachment from pediatric health care professionals (HCPs) are important to reduce concerns and uncertainties of adolescent survivors regarding the transition process and to promote subjective readiness for transition. To gain confidence in the adult healthcare, it is crucial to provide tailored education depending on individual requirements and needs and to build trusting relationships between survivors and adult HCPs.

## 1 INTRODUCTION

In Germany, children diagnosed with cancer survive their initial disease in more than 80%, and the majority will become long‐term survivors.^1^ At the same time, cancer therapies place survivors at an increased risk for developing chronic health complications and late effects. Due to managing chronic conditions and to screen for late‐onset complications that might occur in adulthood, life‐long follow‐up care is necessary.^2^, ^3^ In Germany, young childhood cancer survivors typically continue to attend regular oncological follow‐up care provided by pediatric oncologists until they reach approximately the age of 18.^4^ However, as patients reach adulthood, they are typically referred to family medicine or internal medicine specialists and necessary subspecialists, depending on the specificities of the disease and the patient.^5^ This transition to adult healthcare can be a critical period in the process, especially considering that most children are diagnosed with cancer at an early age and may already have completed their treatment a long time before they turn 18.^6^ Consequently, a significant number of childhood cancer survivors tend to discontinue regular follow‐up care during this time.^7^, ^8^

Compared to adolescents with other chronic diseases (e.g., cystic fibrosis, diabetes, and congenital heart diseases) who often have symptoms at the time of transition, childhood cancer survivors are frequently asymptomatic and in good health when transitioning to adult healthcare. Moreover, if cancer occurred during early childhood, survivors may have limited knowledge of their disease and its treatment.^9^ These factors can contribute to drop‐out from follow‐up care.^10^, ^11^ Additionally, patient factors (e.g., anxiety and self‐management skills), support factors (e.g., family and peers), and system factors (e.g., HCPs, pediatric, and adult healthcare setting) have been identified as potential barriers and/or facilitators to transition.^11^, ^12^ Survivors' attitudes towards pediatric follow‐up care and their experiences with pediatric HCPs can also influence the transition process. For instance, a review has found that a strong bond between survivors, their parents and the pediatric HCPs, along with concerns about ending this connection, can pose potential barriers.^12^ Multiple studies have highlighted knowledge deficits among survivors regarding their primary diagnosis, its treatment, and the risk of potential late effects.^9^, ^13^, ^14^ Transitioning to the adult healthcare system, which requires patients to take more initiative and schedule appointments independently, may be hindered by this lack of knowledge or competence beyond the pediatric oncology setting.^12^, ^15^ In a Swiss study of young adults, approximately one third (36%) of survivors expressed a preference for doctors to communicate with their parents instead of them, and nearly one‐third (28%) indicated that they do not consult a doctor or nurse when they have health concerns.^16^ Therefore, empowering survivors for self‐management and providing them with practical information about the healthcare system are crucial aspects of the transition process, enabling them to make informed medical decisions.^9^, ^12^, ^13^, ^15^, ^17^, ^18^ However, despite the availability of developed and validated transition readiness tools, and a feasibility study demonstrating their easy integration into clinical routines, these tools are not commonly implemented.^12^, ^16^ To enhance the understanding of the transition process and preparation from pediatrics to adult healthcare from the perspective of adolescent cancer survivors can contribute to optimizing current clinical practice and sensitizing healthcare professionals (HCPs) in pediatrics on the most effective ways to prepare patients and their parents for the transition. Thus, the objective of this paper was to investigate two research questions: (a) What attitudes do adolescent survivors have towards pediatric follow‐up care? (b) What are their concerns regarding the transition process?

## 2 METHODS AND ANALYSIS

### 2.1 Design

In this study, we adopted a qualitative research design to gain an in‐depth understanding about pediatric follow‐up care and transition processes.^19^ The interview study is part of a mixed‐methods study on the impact of childhood cancer and adolescent survivors' unmet needs in long‐term follow‐up (LTFU) care. Due to the complexity and richness of the qualitative data, we decided to publish two manuscripts, focusing on different aspects each. The first publication focuses on the burden and support needs of adolescent childhood cancer survivors in long‐term follow‐up care.^20^ The reporting of this study is oriented to the Consolidated Criteria for Reporting Qualitative Research (COREQ).^21^ The study was approved by the Local Psychological Ethics Committee of the Center for Psychosocial Medicine of the University Medical Center Hamburg‐Eppendorf (Study‐ID: LPEK‐0384). Informed consent from all participants and parents' written permission were obtained.

### 2.2 Participants and procedure

Interviews were conducted with adolescent childhood cancer survivors who were attending regular pediatric follow‐up care and prior to their transition into adult healthcare. They were recruited through the department for Pediatric Hematology and Oncology at the Hamburg‐Eppendorf University Medical Center. If they were interested in participation, study material, detailed study information, the informed consent form, and parents' written permission were sent to eligible families by mail. After 6 weeks of non‐response, a friendly phone reminder was given. The inclusion criteria were an age over 14 years at time of the study, a childhood cancer diagnosis, completion of cancer treatment before study participation, and sufficient German language skills. In total, 21 adolescents were interviewed.

### 2.3 Data collection

The authors (JW and VP) conducted the interviews between October 2021 and March 2022, either in person at an office of the university hospital or by telephone. Based on a consecutive sampling approach and considering previous research indicating that saturation can be achieved with a small number (9–17) of interviews, we concluded recruitment once no new aspects were being raised.^19^, ^22^ Before starting the interviews, the interviewers introduced themselves, and participants were verbally informed about the study, data protection, organizational concerns, and their questions were answered. The interview guide focused on participants' experiences with pediatric follow‐up care and their subjective readiness for transitioning into adult healthcare (see Appendix S1). Field notes were taken during the interview. All interviews were audio‐recorded (length: 19–65 min, average 33 min) and transcribed verbatim.

### 2.4 Data analysis

Principles of qualitative content analysis according to Kuckartz were applied using a deductive‐inductive procedure that was data driven and iterative.^23^ The qualitative content analysis software MAXQDA (2022) was used. Initial key categories were generated deductively based on the explorative research questions, the interview guide, and theoretical considerations. Identified codes were reviewed and refined, and subcategories were developed inductively based on the material and through systematic coding of the data. The authors (JW and LI) periodically met to discuss expected and evolving codes, (anchor) quotations, and interpretations. Pre‐existing assumptions and the potential subjectivity of interpretations were also critically reflected within the research group. Based on the finalized coding structure, data were systematically recoded by JW. To ensure intercoder reliability, five interviews (24%) were double‐coded by a member of the research team (DS), and the intercoder reliability was 85%, with consensus for coding reached.

## 3 RESULTS

### 3.1 Sample characteristics

Demographic and medical characteristics of our study sample (*n* = 21) are displayed in Table 1. More male (57%) than female survivors (43%) took part in the study; mean age was 16.7 years (range: 14–20 years). Most participants were diagnosed with leukemia (43%) and had received chemotherapy either alone (62%) or in combination with other treatments (24%). The median age at diagnosis was 5 years (range: 1–17 years).

**TABLE 1:** Adolescent childhood cancer survivors' general and medical characteristics.

Characteristic	*n* = 21 (%)
Gender	
Female	9 (43)
Male	12 (57)
Age, years	Mean = 16.7 (R: 14–20)
14–15	5 (24)
16–20	16 (76)
Nationality	
German	20 (95)
Swiss, living in Germany since childhood	1 (5)
Diagnosis	
Leukemia	9 (43)
Lymphoma	5 (24)
Sarcoma	2 (10)
Brain tumor	4 (19)
Wilm's tumor	1 (5)
Age at diagnosis, years	Median = 5 (R: 1–17)
1–7	13 (62)
8–17	8 (38)
Treatment	
Chemotherapy only	13 (62)
Chemotherapy + bone marrow transplant	1 (5)
Surgery only	1 (5)
Surgery + chemotherapy	3 (14)
Surgery + radiation therapy	2 (10)
Surgery + chemotherapy + radiation therapy	1 (5)

*Note*: Self‐report data.

Derived from the exploratory research questions, two key categories were generated: (a) Survivors' attitudes towards pediatric follow‐up care, which encompasses all formal and emotional aspects of survivors regarding follow‐up care, and (b) their concerns regarding transition from pediatric to adult healthcare, where hindering and facilitating factors for a successful transition occur. A summary definition of each key category can be found in the Appendix S2.

#### 3.1.1 Key category: Follow‐up care

The following findings relate to adolescent childhood cancer survivors' perspective on follow‐up care. The findings were categorized into two subcategories: (1) satisfaction with pediatric follow‐up care focusing on the organizational and content‐related aspects of appointments, and (2) the relationship with HCPs representing survivors' personal positive and negative experiences with HCPs.

##### Subcategory: Satisfaction with pediatric follow‐up care

All childhood cancer survivors reported that they were satisfied or even very satisfied with follow‐up care provided at the pediatric hospital. Appointments were experienced as very relaxed and well‐organized, as survivors reported that they would be seen quickly. Some survivors expressed not only their satisfaction but also their gratitude for the follow‐up care they received. The regular appointments and check‐ups would make them feel safer and less worried about the recurrence of the disease (Table 2, Q1). Nevertheless, survivors expressed a desire for improvement in their follow‐up care. They described a need for more information about support offers, such as self‐help groups or psychotherapy, and for more education from HCPs about their disease, its potential late‐effects, and the organization and structure of follow‐up appointments (Table 2, Q2).

**TABLE 2:** Key category (A): Aspects of follow‐up care from perspective of adolescent childhood cancer survivors; quotations in the text, illustrative quotes and category definitions.

Quotations in the text	Illustrative quote
1. Subcategory: Satisfaction with follow‐up care	
Dissatisfaction/Satisfaction with appointment organization and content	
Q1	“(…) And I am also very grateful that there is follow‐up care. Because if I didn't go to follow‐up care at all, I would worry much more often about whether it [the cancer] might come back, and just because you have to go once a year and have a checkup, you feel more secure when you've had it and the result is negative, that there's nothing suspicious. (…)” (Code 04, female, 18 years old)
Q2	“Except that now and then I was informed a bit too late about some appointments … You have appointments outside the children hospital which you must attend once a year, for example. Me and my mother always knew a little too late. I only went to the doctor after 2 years because I didn't know about it. (…).” (Code 13, male, 18 years old)
2. Subcategory: Relationship with HCPs	
Positive/negative experiences with HCPs	
2.1. Subcategory: Survivors' feeling of being in good hands	
Sense of being well‐cared for in follow‐up care	
Q3	“[…] but in the meantime, when I have the MRI oncology follow‐up appointments, then I almost look forward to it, because I like that. […] also seeing the people again and it's also a feeling of gratitude that it turned out that way. And if they did not exist, and if it hadn't all gone so well, then I would probably be dead now.” (Code 20, male, 20 years old)
2.2. Subcategory: Meeting survivors at eye level	
Survivors' feeling of being treated equally and empathetically by HCPs	
Q4	“Not really. I mean, when I go to appointments or something, they talk to my mom first and then they talk to me. I don't know why it is like that, but that's just the way it is.” (Code 02, female, 15 years old)

*Note*: The quotations in the text were translated from German.

##### Subcategory: Relationship with HCPs

Within the second subcategory, relationship with HCPs, two more subcategories emerged from the data: (1) survivors' feeling of being in good hands, defined as the sense of being well‐cared for in follow‐up care, and (2) meeting survivors at eye level, referring survivors' feeling of being treated equally and empathetically by HCPs.

###### Subcategory: Survivors' feeling of being in good hands

Most survivors reported that they had felt well taken care of at the pediatric hospital and reported that HCPs treated them with respect. For example, survivors reported that they had felt comfortable asking any questions or raising concerns if they were dissatisfied. Additionally, having known the HCPs for a long time had instilled a sense of confidence and reassurance. One survivor even reported that he would look forward to follow‐up appointments and seeing the HCPs again (Table 2, Q3).

###### Subcategory: HCPs meeting survivors at eye level

Survivors expressed that HCPs had met them respectfully and at eye level by explaining everything to them, involving them, and not treating them “like children.” They reported that their individual needs were being met, such as allowing one survivor to see his tumor through a microscope or scheduling appointments in accordance to school demands. Few survivors expressed the impression that HCPs would not meet them at eye level by talking arrogantly or communicating first with their parents (Table 2, Q4).

#### 3.1.2 Key category: Transition process

The following findings relate to the perspective of childhood cancer survivors on the transition process to adult healthcare. Two subcategories emerged under the key category transition process: (1) (subjective) education about the former disease, defined as survivors' own perception about their personal health status and healthcare, and (2) readiness for transition, referring to survivors' perception of their preparedness and willingness to transition from pediatric to adult healthcare.

##### Subcategory: (Subjective) education about the former disease

Four subcategories emerged from the data and relate to the subjective education of adolescents regarding their disease: (1) Knowledge of disease referring to the depth and accuracy of understanding about the former disease and its treatment, (2) awareness of potential late effects pertaining to the understanding and acknowledgement of potential long‐term consequences as a result of the former disease or its treatment, (3) information provider indicating the sources or channels from which survivors receive information/informed themselves about their former disease and potential late effects, and (4) extent of information representing the perceived amount of received information about their former disease and its potential late effects.

###### Subcategory: Knowledge of disease

Survivors were asked about their former childhood cancer disease, the treatment they received, and their age at time of diagnosis. Most participants were able to recall their diagnosis (except for one participant whose father provided the correct answer during the telephone interview), the treatment they received, and at what age they had been diagnosed. A few participants could not recall their treatment, and some were unsure about the exact age at diagnosis and could only provide an approximate age range (Table 3, Q5).

**TABLE 3:** Key category (B): Aspects of the transition process from the perspective of adolescent childhood cancer survivors; quotations in the text, illustrative quotes, and category definitions.

Quotations in the text	Illustrative quote
1. Subcategory: (Subjective) education about the former disease	
Survivors' own perception about personal health status and healthcare	
1.1 Subcategory: Knowledge of disease	
Survivors' depth and accuracy of understanding about the former disease and its treatment	
Q5	“Not so really anymore. […] I can't remember it.” (Code 16, male, 16 years old)
1.2. Subcategory: Awareness of potential late effects	
Survivors' understanding and acknowledgement of potential long‐term consequences resulting from the former disease or its treatment	
Q6	“What is interesting is, I was never informed about any late effects. So, I have no idea if anything that I have could somehow be related to that. I never had that complete clarification, which of course makes you more nervous again and makes you think because you have no idea what really happened or what could happen.” (Code 10, female, 16y)
Q7	“I accept it that way because without chemotherapy I would not be alive anymore and then I rather accept that.” (Code 09, male, 17 years old)
Q8	“Well, you do have the fear, but you just have to keep going.” (Code 06, female, 16 years old)
1.3. Subcategory: Information provider	
Survivors' sources and/or channels from which they receive information/informed themselves about their former disease and potential late effects	
Q9	“I don't think I was told much [about the disease and potential late effects] at the follow‐up, but my parents explained pretty much afterwards.” (Code 03, female, 15 years old)
Q10	“I dealt with it in my spare time. Otherwise from the doctor or anybody I didn't really get much about it. […] I used to watch videos about it, read books. So, I generally dealt with it.” (Code 12, female, 17 years old)
1.4. Subcategory: Extent of information	
Survivors' perceived amount of received information about the former disease and potential late effects	
Q11	“Because sometimes it really bothers me that when I think about it, that I had the disease, but I know quite little about it or that I realize that others know better about it. And I had the disease, but I don't know.” (Code 07, female, 16 years old)
2. Subcategory: (Subjective) readiness for transition	
Survivors' perception of their preparedness and willingness to transition from pediatric to adult healthcare	
Q12	“I really don't have much of an idea where the difference is now, maybe in the therapy or in the follow‐up appointments.” (Code 18, male, 15 years old)
2.1. Subcategory: Support needs	
Subjective support needs to feel better prepared for transition into adult healthcare	
Q13	“Just, like I said, talk to somebody about it [transition], go through the file […] and just get questions answered.” (Code 07, female, 16y)
2.2. Subcategory: Decision‐making	
The way in which and with whom the survivors come to a decision	
Q14	“My Mother. […] But now I make the appointments myself because I'm old enough. But she is still there, and she is also the one who is always called by the doctor and gets the result. And when she is not working, she still accompanies me to the follow‐up appointments. She is still very involved in everything.” (Code 04, female, 18 years old)

*Note*: The quotations in the text were translated from German.

###### Subcategory: Awareness of potential late effects

Regarding potential late effects of their cancer disease and its treatment, most interviewed survivors reported being aware of them. However, there are differences in the scope of information. While one adolescent knew exactly that his fatigue would be one of the late effects of his chemotherapy, a second survivor had not known exactly which late effects could occur. Some survivors also reported that they were never informed about any late effects and mentioned feeling nervous about it (Table 3, Q6). Many of the participants who were aware of potential late effects reported that they were not burdened by the risk for late effects. Reasons reported were confidence in the HCPs, having already survived cancer, currently having no complaints, and accepting late effects as a prize for survival due to chemotherapy (Table 3, Q7). Some participants who were aware of potential late effects mentioned that they were burdened by living with the increased risk for physical late effects and being afraid of late effects. To cope with these fears, they reported that they would try to stay positive, repress negative thoughts and not to worry, even though this might not feel adequate (Table 3, Q8).

###### Subcategory: Information provider

Most adolescents reported that they had received information about their diagnosis, treatment, and potential late effects mainly from their parents. They mentioned that little information was provided by HCPs during the disease, but if so, it was provided in an age‐appropriate manner, such as handing out children's books about cancer or explaining the disease in simple terms (Table 3, Q9). Few survivors mentioned that they had informed themselves by using the internet, watching videos, and reading books (Table 2, Q10).

###### Subcategory: Extent of information

Most participants perceived that they were provided with extensive information including technical terms that were explained to them, they had always known what was coming next, and the information provided was sufficient and detailed. However, some participants described the information about their disease and its treatment as less detailed. While some participants described the amount of information as appropriate, one participant was bothered by the discrepancy between her knowledge about her disease and its consequences and the extent of knowledge of others (Table 3, Q11).

##### Subcategory: (Subjective) readiness for transition

Most survivors expressed that they were not ready for transition as they preferred to continue receiving care in the pediatric healthcare system. Main reasons given were insecurity and a lack of information about the transition process, familiarity with the pediatric hospital, and confidence in their HCPs (Table 3, Q12). However, a few survivors reported feeling prepared for transition, citing reasons such as knowing what to expect, feeling too old for pediatric care, or having confidence in their ability to manage their own healthcare. Within the second subcategory, (subjective) readiness for transition two more subcategories emerged from the data: (1) support needs referring to the perceived need of support to feel better prepared for transition into adult healthcare, (2) decision‐making indicating the way in which and with whom survivors come to a decision.

###### Subcategory: Support needs

When participants were asked about their support needs to feel better prepared for the upcoming transition, they mentioned that they would need more detailed information about the transition process itself and the adult healthcare setting. Additionally, adolescents assumed it was important that their parents would involve them more, for example, in organizational matters (e.g., filling out forms). Survivors also expressed the need for a designated contact person to talk to and ask questions about the transition process (Table 3, Q13).

###### Subcategory: Decision‐making

Most participants reported their parents would be mainly involved in the context of decision‐making. While some survivors reported that their parents would make the decision for them without involving them, some described that their parents had already started to involve them in decision‐making. Few participants reported that they would make decisions on their own, with their parents acting as advisors to weigh options or provide opinions (Table 3, Q14).

## 4 DISCUSSION

The aim of this study was to explore the perspective of adolescent childhood cancer survivors on the pediatric follow‐up care and their concerns regarding their transition process into adult healthcare. By adopting a qualitative research design, we were able to gain an in‐depth understanding about survivors' experiences with the pediatric follow‐up care and their readiness for transition.

Overall, the interviews showed that most adolescents were very satisfied with the pediatric follow‐up care and described a good relationship with their HCPs. Short waiting times, feelings of security due to regular checkups, having the feeling of being in good hands, and the HCPs meeting survivors at eye level were mentioned by the participants. It becomes clear that the relationship between adolescent survivors and HCPs is a crucial factor in satisfaction with follow‐up care as participants who perceived HCPs as arrogant or experienced that HCPs had spoken to their parents first reported less satisfaction in this context.

Our findings are consistent with the results from a meta‐analysis that has shown that patient satisfaction is influenced by psychological, emotional, and relational interactions between patients and HCPs.^24^ Survivors in our study expressed a desire for more information from HCPs about their former disease, potential late‐effects, and psychosocial support offers such as self‐help groups or psychotherapy. Additionally, they expressed a need for more education regarding the organization and structure of follow‐up appointments. Building on previous studies that have examined young adult childhood cancer survivors, we found that even adolescent survivors may report knowledge deficits that are essential for optimal medical self‐management.^25^, ^26^ Knowledge regarding their own cancer history, the risk for potential late effects, and the need for LTFU care have been identified as key factors for a successful transition.^12^ Although most participants in our sample knew about their cancer history and potential late effects, they did not feel ready for transition, felt uncertain and worried. Similar to findings of previous studies,^15^ our results indicate that both awareness and a lack of knowledge about late effects can be associated with uncertainty, fears, and worries.

A study has found that educating survivors about potential late effect can be a balancing act between too little and too much education.^8^ By making increasing efforts to educate survivors about the importance of participation in and adherence to LTFU care, HCPs may heighten worries about secondary tumors and late effects. If survivors have too little education, they may not have many concerns, but they may also be lost to follow‐up.^8^ Rather than excessive education, there might be more opportune times for survivors to receive comprehensive information about their former cancer disease. This becomes also important when it comes to health‐related decision‐making processes. Survivors in the present study were not always actively involved in decision‐making processes, and in some instances, decisions were made exclusively by parents. According to the legislation in Germany, minors up to the age of 18 have the right to express their opinions and their preferences of involvement. They can also decide not to make a decision and determine the extent to which the parents are involved, making the decision together with the children and adolescents. Their level of participation should be considered according to their age and maturity level. The participation of minors in decision‐making processes regarding their treatment and future therapeutic interventions is also an essential right to which they are entitled.^27^ As information needs may vary based on age and other factors,^28^ it is important to provide information that is appropriate for survivors' age and maturity level to further their knowledge according to their cognitive and emotional capacities. As age alone may not be a sufficient indicator of maturity, other factors such as individual maturity and survivors being in a stable phase of the disease process should also be taken into account.^4^, ^29^ The aim of adequately educating and informing survivors is to reduce uncertainty and anxiety, and to ensure informed decision‐making.

The German society of transition recommend training for the patient and, if necessary, their parents/caregivers on relevant aspects of the disease and transition processes.^4^ It is important to provide information early on that corresponds to the survivors' individual needs, such as education on how the transition process takes place and how adult healthcare is organized. However, the question remains as to who in the German healthcare system could take on the role of liaison and mediate between pediatric and adult healthcare. During adolescence, a critical developmental phase with changes at various levels (e.g., physical, emotional, and social),^29^ transitioning to adult healthcare and assuming greater responsibility for their own healthcare, as well as changing healthcare providers, may lead to uncertainty and concerns.^30^ To ensure a successful transition, it is not only necessary to provide knowledge, but also to promote emotional detachment processes of survivors to let them feel ready for the transition. A lack of confidence in adult healthcare and dependence on pediatric HCPs can be significant barriers to a successful transition.^31^ To overcome these barriers and build trusting relationships between survivors and adult HCPs prior to the actual transition process, establishing early interaction between the adolescent survivors and adult HCPs is recommended.^4^ Our results support the guideline recommendations, emphasize their relevance, and demonstrate the importance of adapting already developed interventions (e.g., shared care models) and guidelines according to the context of surviving childhood cancer and to the needs of the individual survivor.^4^, ^12^ It is crucial to integrate them into routine practice.

### 4.1 Study limitations

One limitation of this study is that the diagnosis aspect and the age distribution was not adequate, and they do not represent the actual spectrum of pediatric oncology patients. Significantly more survivors with leukemia participated in the study and the average age of onset was higher than in the broader pediatric oncology population.^6^ A further limitation may have been a self‐selection bias since some survivors may have been less willing or unable to participate in the study.^19^ Furthermore, our results do not allow for clear assumptions about the success of participants' transition, as we interviewed the survivors before the transition process. Additionally, recruitment through the Pediatric Oncology/Hematology Department of the University Medical Center Hamburg‐Eppendorf and the requirement for sufficient German language skills may have further biased our sample. Lastly, telephone interviews with adolescents have both advantages and disadvantages. While they limit non‐verbal communication, hindering the interpretation of emotions and reactions, they offer easy access and flexibility, eliminating the need for travel. Although misunderstandings and biases in communication are possible, adolescents may feel more comfortable expressing themselves openly in familiar surroundings without face‐to‐face interaction with their interviewer, potentially leading to higher‐quality data, especially for sensitive topics.

### 4.2 Clinical implications

The integration of survivors into the organization processes and routines, and the promotion of emotional detachment from pediatric HCPs are important to reduce concerns, fears, and uncertainties of adolescent survivors regarding the transition process and to promote subjective readiness for transition. Moreover, it is essential to provide information in a manner that considers the survivor's age, individual maturity, and whether survivors are in a stable phase of the disease process. To gain confidence in adult healthcare, it is crucial to provide tailored education depending on individual requirements and needs and to build trusting relationships between survivors and adult HCPs.

## 5 CONCLUSION

In the qualitative interviews, it becomes clear that most adolescents were satisfied with the pediatric follow‐up care, including organizational and emotional aspects. However, survivors expressed information needs from HCPs concerning their disease and follow‐up appointments, even though most participants in our sample knew about their cancer history and potential late effects. The results show that most survivors did not feel ready for transition and experienced uncertainty and worry in this context. Our results align with those of previous studies, emphasizing the importance of providing tailored information and support, along with reducing worries and insecurities about adult healthcare, to ensure a successful transition.

## AUTHOR CONTRIBUTIONS

**Jana Winzig:** Data curation (equal); formal analysis (equal); investigation (equal); project administration (equal); visualization (equal); writing – original draft (equal); writing – review and editing (equal). **Laura Inhestern:** Funding acquisition (equal); project administration (equal); resources (equal); supervision (equal); writing – review and editing (equal). **Désirée Sigmund:** Validation (equal); writing – review and editing (equal). **Verena Paul:** Writing – review and editing (equal). **Lesley‐Ann Hail:** Writing – review and editing (equal). **Stefan Rutkowski:** Conceptualization (equal); funding acquisition (equal); resources (equal); writing – review and editing (equal). **Gabriele Escherich:** Conceptualization (equal); funding acquisition (equal); resources (equal); writing – review and editing (equal). **Corinna Bergelt:** Conceptualization (equal); funding acquisition (equal); methodology (equal); resources (equal); supervision (equal); writing – review and editing (equal).

## FUNDING INFORMATION

We acknowledge financial support from the Open Access Publication Fund of UKE ‐ Universitätsklinikum Hamburg‐Eppendorf and DFG ‐ German Research Foundation. This study was funded by Hamburg Cancer Society and the Sponsoring Association of the Hamburg Pediatric Cancer Center and was conducted within the endowed professorship for healthcare research in pediatric rare diseases (Kindness for Kids professorship) which had neither influence on the study design nor the outcome of this study.

## CONFLICT OF INTEREST STATEMENT

The authors have no conflicts of interests to disclose.

## Supporting information


Appendix S1.

Appendix S2.

## Data Availability

Data can be obtained on request from the corresponding author.
