# Supplementary material for: Exploring the perspective of adolescent childhood cancer survivors on follow‐up care and their concerns regarding the transition process—A qualitative content analysis
Source: Cancer Med. 2024 May 16;13(10):e7234. doi: 10.1002/cam4.7234 (PMC11097248; doi:10.1002/cam4.7234)
Supplement: Supplementary file 1 — Appendix S1. [file CAM4-13-e7234-s001.docx]

| **Key Kategory** | **KEY QuestionS** |
| --- | --- |
| **follow-up care** | - How satisfied are you with your follow-up care? - Are there any aspects of the follow-up care that you believe could have been improved? - To what extent have you been educated about your disease (e.g., medication, assessment of changes in your health situation, co morbidities, frequencies of follow-up appointments)? - **If this has not been addressed yet**: Who provided you with information about your cancer disease? - Were you able to comprehend the information effectively? - Are you aware that long-term effects can result from your childhood cancer disease and its treatment? - **If yes**, how do you feel about this? |
| **Transition Process** | Starting at a specific age, pediatric oncologists are no longer responsible anymore, and long-term follow-up care is handled by adult doctors. I would now like to learn more about your experiences during the transition from pediatric to adult care.Do you feel well-prepared for the transition from pediatric to adult medicine (and with the self-organization and self-responsibility regarding your condition)?**If not,** why not? What type of support do you wish you had to feel more prepared?Do you have any worries, anxieties, or insecurities regarding your transition to adult care?If you could choose freely, would you opt to transition to adult care?What role does your family, especially your parents, play concerning your disease?Has your parents’ role changed with regard to your disease in the past few years?Who makes the decision for your healthcare provision?**If it’s your parents,** do you feel that they include you in these decisions?What about appointments with doctors and medical staff: Do you feel that they communicate with you on an equal footing and include you?Do you feel taken seriously by health care professionals (HCPs) (doctors, nurses, etc.)?Do you feel secure enough to make important decisions for yourself, or are you rather insecure in that regard? |
