# Supplementary material for: Exploring the perspective of adolescent childhood cancer survivors on follow‐up care and their concerns regarding the transition process—A qualitative content analysis
Source: Cancer Med. 2024 May 16;13(10):e7234. doi: 10.1002/cam4.7234 (PMC11097248; doi:10.1002/cam4.7234)
Supplement: Supplementary file 2 — Appendix S2. [file CAM4-13-e7234-s002.docx]

| **Key category** | **Definition** |
| --- | --- |
| 1. Survivors’ attitudes towards pediatric follow-up care | The attitudes of childhood cancer survivors towards pediatric follow-up care are defined as their overall perception of the care they receive, their satisfaction or dissatisfaction with it, and any emotional or practical considerations they may have related to their follow-up care. |
| 1. Concerns regarding the transition process | Concerns regarding the transition process includes all considerations, anxieties and worries that arise in the context of transition from pediatric into adult healthcare and may relate to different domains such as support needs, autonomy and self-advocacy, and medical continuity. |
